# ExoU Activates NF-κB and Increases IL-8/KC Secretion during *Pseudomonas aeruginosa* Infection

**DOI:** 10.1371/journal.pone.0041772

**Published:** 2012-07-26

**Authors:** Carolina Diettrich Mallet de Lima, Teresa Cristina Calegari-Silva, Renata Meirelles Santos Pereira, Sabrina Alves de Oliveira Lima Santos, Ulisses Gazos Lopes, Maria-Cristina Maciel Plotkowski, Alessandra Mattos Saliba

**Affiliations:** 1 Departamento de Microbiologia, Imunologia e Parasitologia, Universidade do Estado do Rio de Janeiro, Rio de Janeiro, Rio de Janeiro, Brazil; 2 Laboratório de Parasitologia Molecular, Instituto de Biofísica Carlos Chagas Filho, Universidade Federal do Rio de Janeiro, Rio de Janeiro, Rio de Janeiro, Brazil; University of North Carolina at Chapel Hill, United States of America

## Abstract

ExoU, a *Pseudomonas aeruginosa* cytotoxin injected into host cytosol by type III secretion system, exhibits a potent proinflammatory activity that leads to a marked recruitment of neutrophils to infected tissues. To evaluate the mechanisms that account for neutrophil infiltration, we investigated the effect of ExoU on IL-8 secretion and NF-κB activation. We demonstrate that ExoU increases IL-8 mRNA and protein levels in *P. aeruginosa*-infected epithelial and endothelial cell lines. Also, ExoU induces the nuclear translocation of p65/p50 NF-κB transactivator heterodimer as well as NF-κB-dependent transcriptional activity. ChIP assays clearly revealed that ExoU promotes p65 binding to NF-κB site in IL-8 promoter and the treatment of cultures with the NF-κB inhibitor Bay 11-7082 led to a significant reduction in IL-8 mRNA levels and protein secretion induced by ExoU. These results were corroborated in a murine model of pneumonia that revealed a significant reduction in KC secretion and neutrophil infiltration in bronchoalveolar lavage when mice were treated with Bay 11-7082 before infection with an ExoU-producing strain. In conclusion, our data demonstrate that ExoU activates NF-κB, stimulating IL-8 expression and secretion during *P. aeruginosa* infection, and unveils a new mechanism triggered by this important virulence factor to interfere in host signaling pathways.

## Introduction

ExoU, a *Pseudomonas aeruginosa* protein injected into host cytosol by the type III secretion system, plays an important role in the establishment and invasiveness of acute infections [1], [2]. The ExoU effects, which include cytotoxicity for a variety of cell types and a potent stimulation of inflammatory response, are mainly mediated by its phospholipase A_2_ (PLA_2_)-like activity that leads to the release of high amounts of arachidonic acid from host cell membranes [3]. This proinflammatory effect can be clearly observed in experimental models of pneumonia that show an intense inflammatory infiltrate rich in neutrophils in lungs of mice infected with the ExoU-producing PA103 *P. aeruginosa* strain, but not in lungs of mice infected with the ExoU-deficient mutant PA103Δ*exoU* [4].

Although neutrophil recruitment is a complex mechanism that depends on a variety of soluble and cell-associated factors, the chemokine Interleukin 8 (IL-8) represents one of the most important products involved in this event. IL-8 is produced at basal levels in physiologic conditions, but proinflammatory stimuli can induce IL-8 secretion by a number of pathways. Among the transcriptional regulators of IL-8, an essential role is conferred to NF-κB, a transcriptional factor found in cytoplasm as homo or heterodimer of Rel family proteins (c-Rel, p65, RelB, p50 and p52) that is translocated to nucleus after cell stimulation. Once in nucleus, NF-κB binds to specific promoter sequences of a large number of target genes, which are involved in diverse cellular responses, including inflammation [5].

To address whether the neutrophil recruitment observed during infection with the ExoU-producing strain is due to NF-κB activation and the consequent IL-8 induction, we investigated the effect of ExoU in *P. aeruginosa*-infected human epithelial respiratory and microvascular endothelial cell cultures, as well as in a murine model of pneumonia. Our data provide novel evidences on the importance of ExoU as a potent NF-κB activator and clarify the role of this transcriptional factor in the inflammatory process during infection by ExoU-producing *P. aeruginosa* strains.

## Results

### ExoU PLA_2_ activity increases IL-8 mRNA and secretion

**Figure 1:**
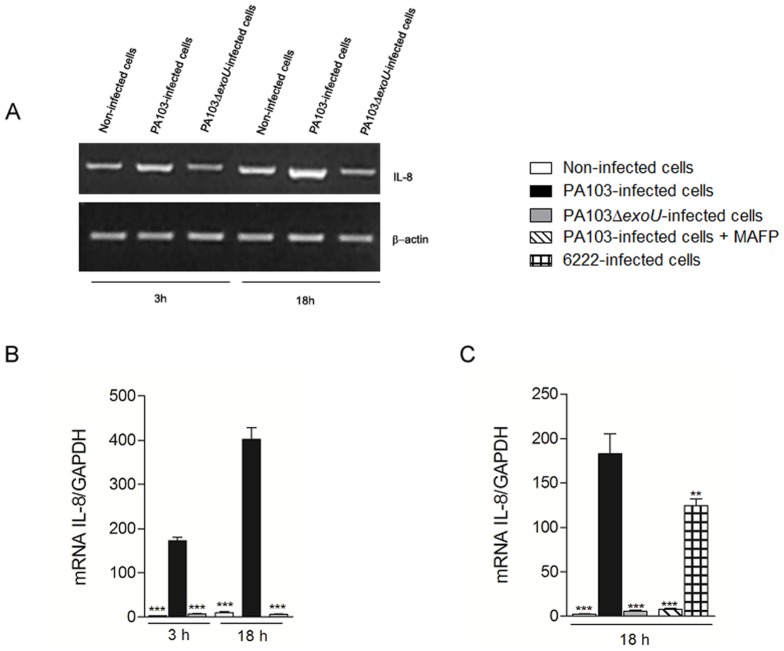
ExoU increases IL-8 mRNA in A549 cells. In (A), representative agarose gels obtained from three different semi-quantitative RT-PCR assays carried out in duplicate. In (B) and (C), graph represents the means ± SEM of values obtained by three different Real Time qRT-PCR assays performed in triplicate. **p<0.01 or ***p<0.001 when the values obtained from cultures infected with the ExoU-producing PA103 strain were compared with those obtained from the other cultures.

To assess the effect of ExoU on IL-8 expression by *P. aeruginosa*-infected airway epithelial cells, A549 cultures were infected for different periods of time by the ExoU-producing PA103 strain or its isogenic mutant, PA103Δ*exoU*, to investigate IL-8 mRNA levels by semi-quantitative and Real Time RT-PCR. Our results showed a significant increase of IL-8 mRNA levels in PA103-infected cultures in comparison with cultures infected with PA103Δ*exoU* and non-infected cultures, and revealed that, although this induction has started as soon as 3 hours post-infection and remained until 18 hours post-infection, more robust differences could be detected in the later period. Moreover, IL-8 stimulation depended on ExoU PLA_2_ activity since treatment of PA103 with the PLA_2_ inhibitor MAFP before A549 infection reduced IL-8 mRNA to the levels detected in PA103Δ*exoU*-infected cultures. Finally, when IL-8 mRNA levels from cells infected with the *exoU*+ clinical strain 6222 were assessed, we observed that both *exoU*+ strains significantly induced IL-8 expression, although PA103 has led to higher mRNA levels, probably due to its stronger ExoU secretion (Fig. 1).

**Figure 2:**
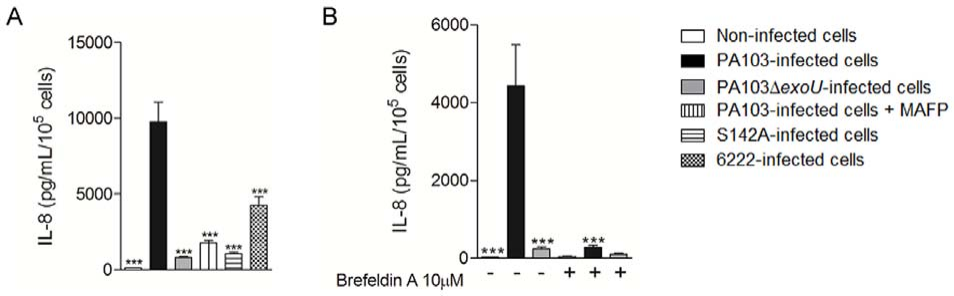
ExoU PLA_2_ activity stimulates IL-8 secretion by *P. aeruginosa*-infected A549 cultures. In (A) and (B), cells were infected with the different *P. aeruginosa* strains for 21 hours and the concentrations of IL-8 in supernatants were assessed by ELISA. In (B), Brefeldin A was added or not to the gentamicin-containing culture medium. The graphs show the means ± SEM of three assays performed in quadruplicate. ***p<0.001 when the values obtained from untreated PA103-infected cultures were compared with those from the other cultures.

To ascertain whether the ExoU-mediated IL-8 mRNA induction was accompanied by IL-8 release, we evaluated the A549 cell culture supernatants by ELISA. These assays showed that infection by both *exoU*+ strains led to a higher detection of IL-8 in cell supernatants, although, once again, PA103 has induced significantly more IL-8 release than the clinical strain. In addition, IL-8 release depended on ExoU PLA_2_ activity since both treatment of PA103 with MAFP and infection with PA103ΔUT/S142A, an *exoU*-deficient mutant complemented with site-directed mutated *exoU* in serine catalytic motif [6], significantly reduced IL-8 concentration in supernatants. Given that PA103 is cytotoxic for a variety of cell types, including A549 epithelial cells (unpublished data), we next evaluated whether the higher amount of IL-8 in supernatants of PA103-infected cells had been due to cell lysis or had resulted from the active secretion by A549. The analysis of the supernatants from cells treated with Brefeldin A, a compound that inhibits IL-8 secretion [7], dramatically reduced the ExoU-dependent IL-8 release (Fig. 2). This result proved that the increased concentration of IL-8 detected in PA103-infected cell supernatants depended on ExoU-induced IL-8 synthesis/secretion, rather than on cell lysis.

### ExoU promotes NF-κB activation in PA103-infected airway epithelial cells

**Figure 3:**
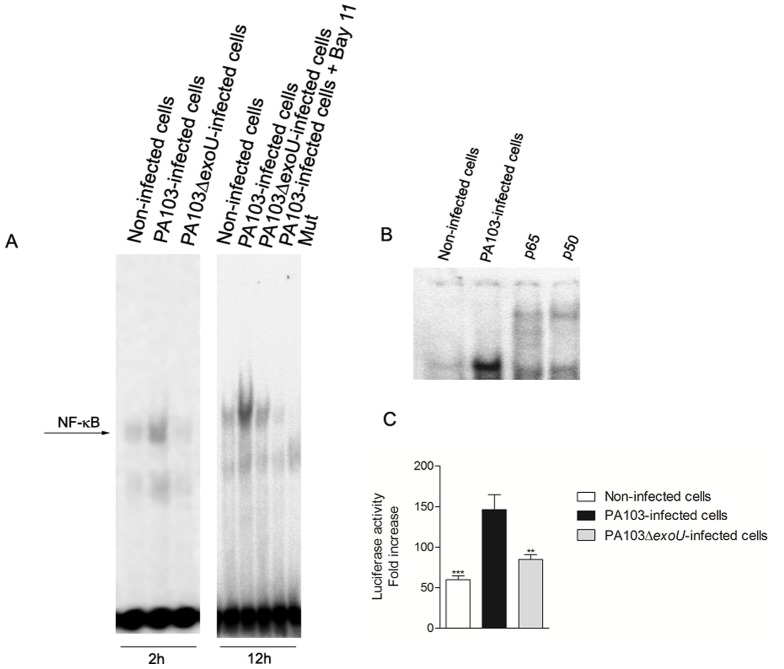
ExoU promotes p65/p50 nuclear translocation and NF-κB-dependent transcriptional activity in *P. aeruginosa*-infected A549 cells. In (A), representative EMSAs showing NF-κB nuclear translocation after 2 and 12 hours of *P. aeruginosa* infection or treatment with culture medium. To evaluate NF-κB inhibition by Bay 11-7082, some cultures were treated with 10 µM Bay 11-7082, 1 hour prior PA103 infection. As a control for non-specific interactions, nuclear extracts from PA103-infected cells were also incubated with a probe mutated in a single nucleotide (Mut). In (B), extracts obtained from PA103-infected cells were supershifted with specific antibodies against NF-κB subunits, p50 and p65. In “Non-infected cells” and “PA103-infected cells” lanes, antibodies were not added. EMSA and supershift assays were performed in triplicate. In (C), the graph shows the luciferase activity (in arbitrary values) of whole-cell lysates obtained from A549 cultures transfected with p6κB-LUC and pRL-CMV plasmids and then infected with *P. aeruginosa* strains or treated with cultured medium for 24 hours. Data represent means ± SEM of three different assays carried out in quadruplicate. **p<0.01 and ***p<0.001 when the values obtained from PA103Δ*exoU*-infected cultures and non-infected cultures, respectively, were compared with those obtained from PA103-infected cultures.

Since the main transcriptional activator of IL-8 expression is NF-κB, we next investigate the ability of ExoU to promote NF-κB nuclear translocation and NF-κB-dependent transcriptional activity. As shown by electrophoretic mobility shift assay (EMSA), infection for 2 and 12 hours with PA103, but not with PA103Δ*exoU*, induced a remarkable NF-κB nuclear translocation, which was abrogated by Bay 11-7082 pre-treatment. A more detailed analysis by supershift revealed that PA103 infection changed the p65/p50 complex into a higher mobility complex, indicating that ExoU induces nuclear translocation of this transactivator heterodimer. Moreover, luciferase reporter assays showed the ability of ExoU to induce NF-κB-dependent transcriptional activity in target genes, since it was observed a significant increase of luciferase activity in PA103-infected cultures when compared to non-infected or PA103Δ*exoU*-infected cultures (Fig. 3).

### IL-8 expression induced by ExoU depends on NF-κB

**Figure 4:**
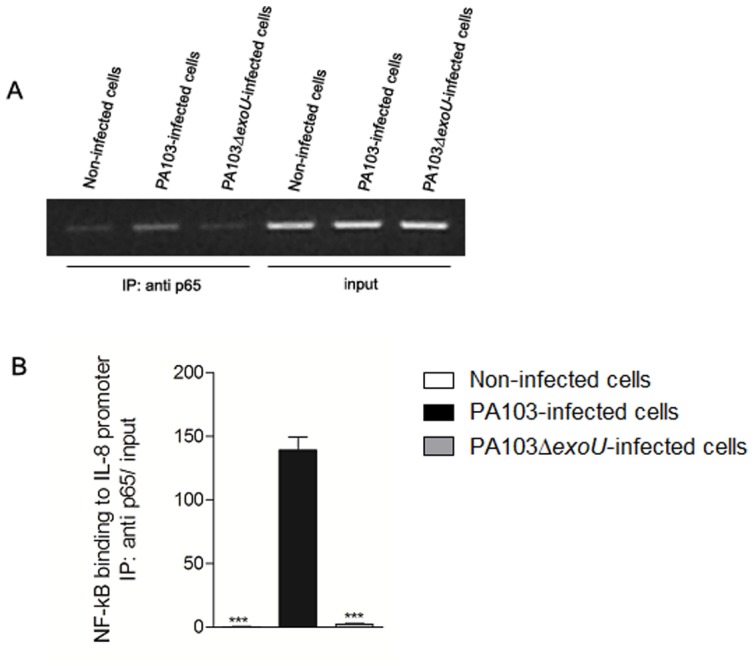
ExoU induces NF-κB binding in IL-8 promoter of A549 cells. In (A), representative agarose gel showing PCR products of DNA obtained after chromatin immunoprecipitation with anti-p65 antibody (IP:anti-p65) or non-immunoprecipitated DNA (input) amplified with primers specific for NF-κB site in IL-8 promoter. In (B), graph shows the means ± SEM of values obtained by Real Time PCR of three different ChIP assays. ***p<0.001 when the values obtained in non-infected cultures or cultures infected with the ExoU deficient strain were compared with those obtained in cultures infected with the ExoU-producing strain for 14 hours.

The ExoU-induced NF-κB nuclear translocation is accompanied by its binding in the promoter region of the IL-8 gene, as revealed by chromatin immunoprecipitation (ChIP) assays that showed a significant increase of p65 binding to NF-κB site in IL-8 promoter when A549 cells were infected with the ExoU-producing PA103 strain. In contrast, infection with PA103Δ*exoU* mutant led to a low NF-κB binding that did not differ from the observed after treatment of control cells with culture medium (Fig. 4).

**Figure 5:**
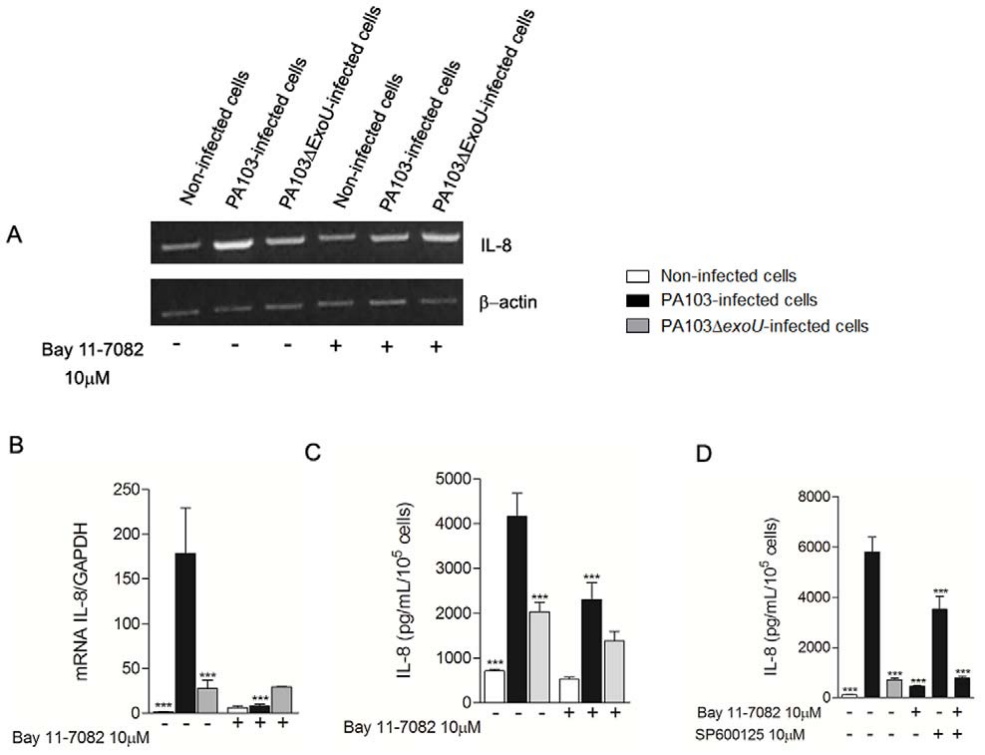
Inhibition of NF-κB reduces IL-8 expression and secretion induced by ExoU in *P. aeruginosa*-infected A549 cultures. In (A), representative agarose gels of RT-PCR assays in which A549 cells were treated or not with 10 µM Bay 11-7082, 1 hour prior infection with PA103 or PA103Δ*exoU* strains or treatment with culture medium (non-infected cells) for 18 hours. In (B), graph shows the means ± SEM of values obtained by three Real Time qRT-PCR assays. In (C), IL-8 concentrations detected in supernatants of cultures pretreated or not with 10 µM Bay 11-7082 for 1 hour and then exposed for 21 hours to PA103, PA103Δ*exoU* or culture medium, as assessed by ELISA. In (D), IL-8 concentrations, as detected by ELISA, in supernatants of cultures pretreated or not with 10 µM Bay 11-7082 and/or 10 µM SP600125 for 1 hour and then exposed for 21 hours to bacterial strains or culture medium. In (C) and (D), data represent means ± SEM of three assays performed in quadruplicate. ***p<0.001 when the values obtained from untreated PA103-infected cultures were compared with those from the other cultures.

To confirm that the ExoU-mediated NF-κB activation is responsible for the higher IL-8 expression, assays were performed with cells treated with the NF-κB inhibitor Bay 11-7082 before infection. Semi-quantitative and Real Time RT-PCR showed that treatment with Bay 11-7082 completely abolished the increase of IL-8 mRNA levels induced by ExoU, whereas ELISA showed that NF-κB inhibition before infection with PA103 strain significantly reduced IL-8 secretion in culture supernatants. Since Cuzick *et al*. 2006 [8] reported a significant reduction in ExoU-mediated IL-8 release by human bronchial epithelial cells from line 16HBE14o- after treatment with the JNK inhibitor SP600125, we next evaluated the effect of this compound in IL-8 secretion by A549 cells. Our results showed that, although treatment with SP600125 reduced IL-8 secretion, the concentration of IL-8 detected in supernatants of cells simultaneously treated with SP600125 and Bay 11-7082 did not differ from the concentration detected in supernatants of cells only treated with Bay 11-7082, suggesting that NF-κB is the main pathway of activation of IL-8 expression in A549 cells infected with ExoU-producing *P. aeruginosa* (Fig. 5).

### Activation of NF-κB and induction of IL-8 by ExoU also occur in capillary endothelial cells

**Figure 6:**
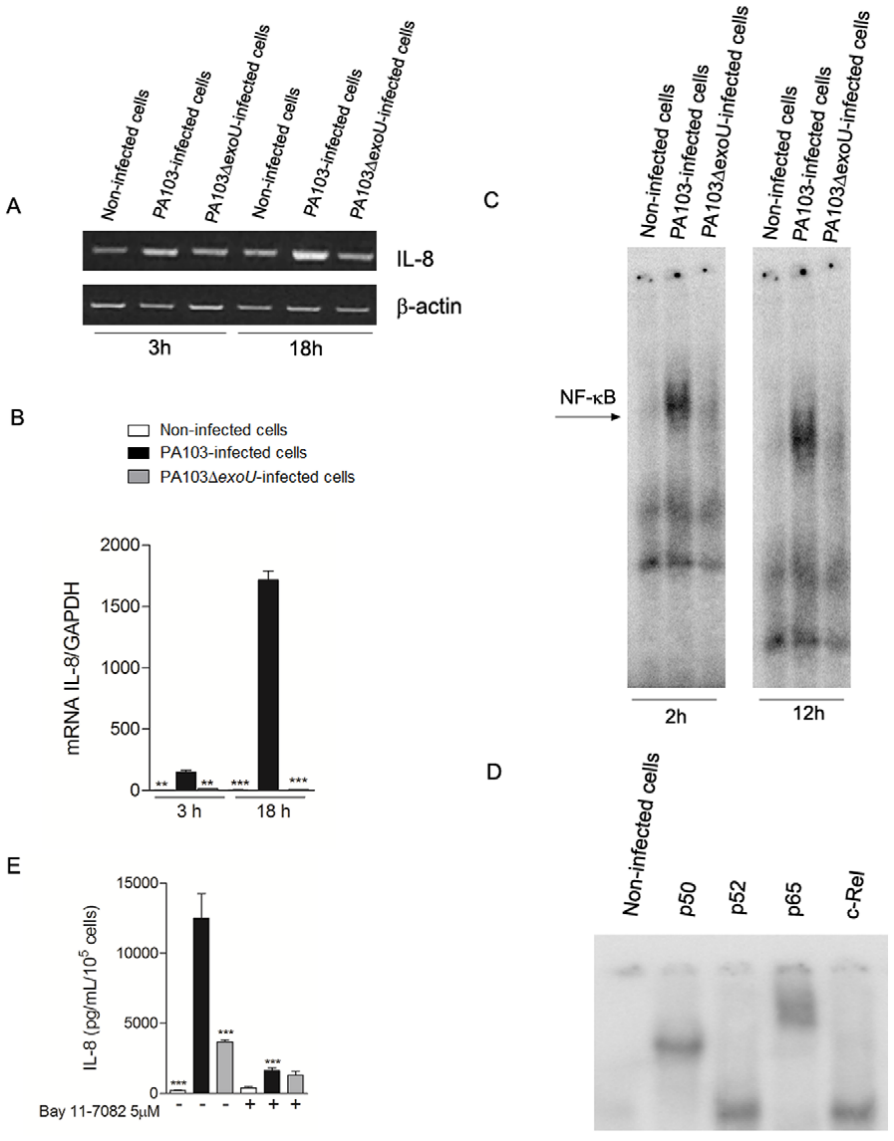
ExoU activates p65/p50 NF-κB and increases IL-8 expression and secretion in HMEC-1 capillary endothelial cells. In (A), representative agarose gels of three different semi-quantitative RT-PCR assays carried out in duplicate. In (B), graph shows the means ± SEM of values obtained in three Real Time qRT-PCR assays. **p<0.01 and ***p<0.001 when the values obtained from PA103Δ*exoU*-infected cultures and non-infected cultures, respectively, were compared with those obtained from cultures infected with the ExoU-producing strain. In (C), representative EMSAs showing NF-κB nuclear translocation after 2 and 12 hours of *P. aeruginosa* infection or treatment with culture medium. In (D), extracts obtained from PA103-infected cells were incubated with specific antibodies for NF-κB subunits, p50, p52, p65 or cRel, but only supershifted with p50 and p65. In non-infected cells, antibodies were not added. EMSA and supershift assays were performed in triplicate. In (E), IL-8 concentrations, detected by ELISA, in supernatants of cultures pretreated or not with 5 µM Bay 11-7082, for 1 hour, and exposed to PA103, PA103Δ*exoU* or culture medium, for 21 hours. The graph represents the means ± SEM of two assays performed in quadruplicate and shows ***p<0.001 when the values obtained from untreated PA103-infected cultures were compared with those from the other untreated cultures or with those from Bay 11-7082-treated cultures infected with the ExoU-producing strain.

Since lungs are the main portal of entry of *P. aeruginosa* bacteremia and ExoU seems to play an important role in invasive infections, we then investigated whether NF-κB activation and IL-8 secretion induced by ExoU were restricted to airway epithelial cells or could also occur in microvascular endothelial cells. As shown in Fig. 6, infection of HMEC-1 cells with the ExoU-producing strain led to increased IL-8 mRNA levels and p65/p50 nuclear translocation. Moreover, HMEC-1 cultures infected with the ExoU-producing PA103 strain were able to secrete significantly more IL-8 than cultures infected with PA103Δ*exoU* or cultures treated with Bay 11-7082 before PA103 infection.

### NF-κB activation by ExoU induces KC secretion in bronchoalveolar lavage fluids (BALF) and is responsible for the robust neutrophil infiltrate in mice airways

**Figure 7:**
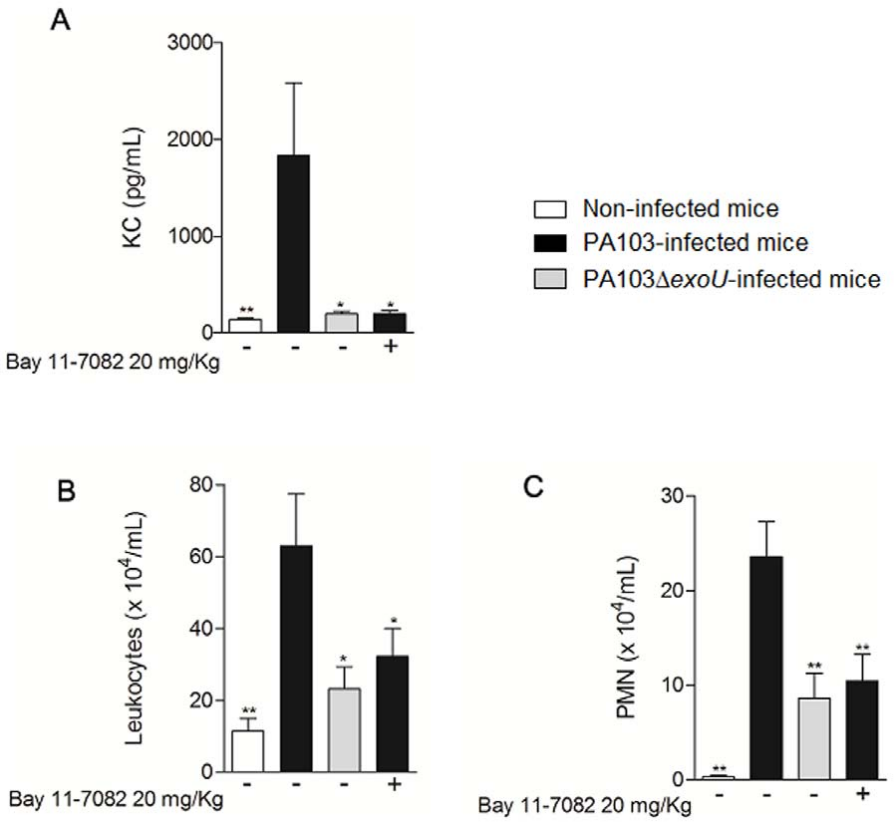
ExoU induces NF-κB-dependent KC secretion and neutrophil infiltration in mice airways at 24 hours post-infection. In (A), concentrations of KC assessed by ELISA, in (B) total leukocytes and in (C) neutrophils (PMN), both assessed by light microscopy, in BALF of mice pretreated or not with Bay 11-7082 at 20 mg/Kg, for 1 hour, and inoculated intratracheally with PA103, PA103Δ*exoU* or saline. The graphs show the mean values ± SEM of three independent assays (n = 16 for each group). *p<0,05 or **p<0.01 when the values obtained in untreated mice infected with PA103 strain were compared with the values obtained in the other groups.

In a mouse model of acute pneumonia, ExoU induced a substantial secretion of KC that was completely abolished when mice were treated with the NF-κB inhibitor Bay 11-7082 before infection. More importantly, KC inhibition by Bay 11-7082 was accompanied by a significant reduction of white blood cells and particularly neutrophils, supporting the essential role of NF-κB in ExoU proinflammatory activity (Fig. 7).

## Discussion

ExoU, a virulence factor with recognizable importance in invasive acute infections, stimulates a proinflammatory response that is characterized, among other events, by a robust neutrophil infiltrate [4], [9]. The early recruitment of neutrophils is a crucial mechanism of defense against *P. aeruginosa*, as proved by the increased mortality of mice submitted to neutrophil depletion before infection and the higher disease severity of neutropenic patients [10]–[12]. However, excessive and continuous neutrophil infiltration and activation is a harmful event because highly reactive intermediates and degradative enzymes secreted by these cells accumulate in tissues, promoting organ damage that could facilitate bacterial spread. Furthermore, ExoU kills neutrophils [13], releasing intracellular products that induce even more inflammation and injury. Hence, by recruiting and killing neutrophils, ExoU could interfere with immune response, promoting a local tissue damage that would favor bacterial dissemination.

The mechanisms regulated by ExoU that lead to neutrophil infiltration are still poorly understood and, although *P. aeruginosa* stimulates ICAM-1 expression in endothelial cell surface, there is no evidence of ExoU contribution to ICAM-1 up-regulation [14]. Here, we show that the ExoU-mediated neutrophil recruitment results, at least in part, from the higher IL-8 expression due to the ExoU-dependent NF-κB activation. This finding is of special interest because NF-κB is a transcriptional factor that regulates a number of genes involved in inflammatory and immune responses and can represent a potential target in *P. aeruginosa*-host interactions.

Although IL-8 stimulation by ExoU in cell culture assays has been previously reported [8], [15], induction of IL-8 expression had only been related to AP-1 pathway. In fact, IL-8 expression could simultaneously be regulated by multiple transcriptional factors and, in a model of chronic infection during cystic fibrosis, a non-producing ExoU *P. aeruginosa* strain stimulated IL-8 secretion through a complex mechanism that included activation of NF-κB, AP-1, NF-IL6, CHOP and CREB [16]. However, in our model, the treatment of cells with a combination of JNK and NF-κB inhibitors did not reduce IL-8 secretion even more than the observed in cells treated with NF-κB inhibitor only. Furthermore, the ExoU-mediated activation of NF-κB pathway is probably a common event since our data show that it occurs in both epithelial and endothelial cells. More importantly, we show that the ExoU-mediated KC secretion and neutrophil recruitment during acute pneumonia was profoundly affected by NF-κB inhibition, which demonstrate the central role of this transcriptional factor in the proinflammatory response induced by ExoU.

Additionally, IL-8 transcription is affected by modulation of epigenetic patterns that regulates chromatin opening and, consequently, the access of transcription factors to the promoter region. A number of studies showed that bacteria can regulate IL-8 gene by inducing or blocking histone modifications, such as histone phosphorylation, methylation or acetylation [17]–[20] Since the transcriptional activation domain of p65 interacts with histone acetyltransferases [21], ExoU activation of NF-κB could induce epigenetic alterations that would contribute to the increased expression of IL-8. The ability of ExoU or other *P. aeruginosa* PAMPs to induce histone modifications during infection of host cells by ExoU-producing strains could represent a promising field of research that remains to be explored.

Bacterial products can bind to cellular receptors, such as *P. aeruginosa* flagellin that binds to TLR5, and directly activate NF-κB, leading to increased IL-8 expression [22], [23]. On the other hand, virulence factors generate a number of cellular responses and lead to the secretion of host products that in turns can activate NF-κB and induce IL-8 secretion. This is an interesting question that remains to be solved. ExoU directly activates NF-κB or this effect is resulted from products generated by ExoU-injected cells that could act in all cells of the neighborhood?

Similar to other PLA_2_ enzymes, ExoU acts in host membranes phospholipids, releasing free arachidonic acid and lysophospholipids, which induce a number of pathways that lead to NF-κB activation. We have previously reported that ExoU, probably by arachidonic acid oxidation or stimulus of NADPH oxidase, causes oxidative stress in *P. aeruginosa*-infected cells [24]. Oxidative stress is widely considered a potent NF-κB activator and it was shown to drive NF-κB-dependent IL-8 expression in a variety of studies [25]–[28]. Additionally, free arachidonic acid is also metabolized by COX-2, leading to higher PGE_2_ secretion in ExoU-injected cells [4], [15]. Since PGE_2_ can activate NF-κB and induce IL-8 expression in epithelial endometrial cells, this mechanism could represent an interesting via to be explored as well [29]. Besides arachidonic acid, infection with ExoU-expressing *P. aeruginosa* strains also lead to release of lysophospholipids, which can be acetylated to produce platelet activating factor (PAF), a potent inflammatory lipid. In fact, we have previously demonstrated that ExoU induces the production of PAF [30], and this compound is known to be able to activate NF-κB and stimulate IL-8 secretion [31]. Finally, diverse other products generated in response to ExoU could activate NF-κB or sustain this activation, such as TNF-α [30] that classically activates NF-κB upon binding to TNFR family members [32].

In conclusion, our study showed that NF-κB is an important target explored by ExoU to modulate host response. Regulation of NF-κB pathway could be used to control the inflammatory response and avoid the injury provoked by the excessive neutrophil infiltration and the products secreted by these cells or released during the ExoU-induced cell death. Moreover, by activating NF-κB, ExoU probably regulates a wide range of genes that participate of diverse cellular processes, which turns this pathway an attractive focus to be investigated. Currently, further studies are being performed in our laboratory to identify the signaling pathways that lead to NF-κB activation by ExoU.

## Materials and Methods

### Bacterial strains

All experiments were performed with PA103 strain and its mutant PA103Δ*exoU* [4]. To evaluate the ExoU PLA_2_ activity, PA103 was treated with the PLA_2_ inhibitor MAFP at 100 µM for 30 minutes before cell infection and/or cultures were infected with the mutant PA103ΔUT/S142A, which possess a site-specific mutation from serine to alanine at aminoacid 142 that inactivates PLA_2_ activity [6]. The clinical strain 6222, isolated from the blood of a patient with respiratory infection, in which the *exoU* gene was detected by PCR, using the primers 5′- CTT CAG GGC AAG GTC TCG -3′ (sense) and 5′- CTT GCG TGT CTC GTC GC -3′ (antisense), and confirmed by sequencing, was used to compare the results obtained with the laboratory strains.

### Cell infection

Human airway epithelial cells from A549 line (purchased from the Rio de Janeiro Cell Bank, BCRJ 0033), and dermal capillary endothelial cells from HMEC-1 line [33] (kindly provided by Dr. Veronica Morandi, State University of Rio de Janeiro, Brazil) were cultured, respectively, in F12 nutrient culture medium (Invitrogen) containing 10% fetal calf serum (FCS) and antibiotics or MCDB-131 medium (Sigma-Aldrich) with 10% FCS, 10 ng/ml of EGF (Sigma), 1 µg/ml of hydrocortisone (Sigma) and antibiotics. Cells were infected with *P. aeruginosa* strains at MOI of 100, centrifuged (1,000×g for 10 min) to favor the close contact between bacteria and host cells and incubated for 1 h at 37°C. Control cultures were exposed to culture medium only. To reduce bacterial challenge and assess host response in later periods, cell cultures were treated with gentamicin at 300 µg/mL to kill extracellular bacteria and incubated for additional time.

To evaluate the mechanism of IL-8 release, 10 µM Brefeldin A, an inhibitor of intracellular protein transport and protein secretion, were added to the gentamicin-containing medium. To assess the role of NF-κB and JNK pathways in IL-8 induction, cells were treated with the NF-κB inhibitor Bay 11-7082 at 10 µM (A549) or 5 µM (HMEC-1) and/or with the JNK inhibitor SP600125 at 10 µM, 1 hour before infection. Brefeldin A, Bay 11-7082 and SP600125 were purchased from Enzo Life Sciences.

### Semiquantitative and Real Time RT-PCR

A549 and HMEC-1 cells infected for 3, 6 or 18 h were trypsinized, washed and total RNA was isolated using the Qiagen Rneasy kit. cDNA was synthesized from 3 µg of total RNA by reverse transcription with the SuperScript™ III First-Strand synthesis system for RT-PCR (Invitrogen), diluted (1∶200) and amplified by PCR under the following conditions: denaturation at 94°C for 2 min and 23 (β-actin) or 31 (IL-8) cycles of denaturation at 94°C for 45 sec, annealing at 52°C (β-actin) or 58°C (IL-8) for 45 sec, and extension at 72°C for 45 sec. An additional extension step of 5 min at 72°C was carried out after the last cycle. Primers used in β-actin reactions were 5′- CCT CGC CTT TGC CGA TCC -3′ (sense) and 5′- GGA TCT TCA TGA GGT AGT CAG TC -3′ (antisense), whereas primers used in IL-8 reactions were 5′- GAG AGT GAT TGA GAG TGG ACC – 3′ (sense) and 5′ – AGC AGA CTA GGG TTG CCA GA – 3′ (antisense). PCR products were subjected to electrophoresis in a 1% agarose gel in TBE buffer and analyzed densitometrically using LabImage software (Kaplan GmbH). Real-time qRT-PCR was performed in the Applied Biosystems StepOne detection system (Applied Biosystems) using GoTaq qPCR Master Mix (Promega). Experimental qRT-PCR data were normalized by GAPDH as an endogenous control. Primers used in IL-8 reactions were 5′- AAG AAA CCA CCG GAA GGA AC – 3′ (sense) and 5′- AGC ACT CCT TGG CAA AAC TG – 3′ (antisense), whereas primers used in GAPDH reactions were 5′- TGC ACC ACC AAC TGC TTA GC - 3′ (sense) and 5′ – GGC ATG GAC TGT GGT CAT GAG – 3′ (antisense). All expression ratios were determined by the relative gene expression ΔΔ*Ct* method *via* StepOne 2.0 software 2.0 (Applied Biosystems).

### EMSA and supershift for NF-κB

Nuclear extracts from A549 and HMEC-1 cells were obtained as previously described [34]. EMSA was performed by incubating 3 µg of nuclear protein extract with 50,000 CPM of ^32^P-end-labeled double-stranded NF-κB consensus oligonucleotide (Santa Cruz Biotechnology) for 30 min at 25°C. The binding mixture also included 1 µg of poly(dI-dC):poly(dI-dC) in binding buffer (10 mM HEPES, pH 7.9; 4% glycerol; 1 mM DTT; 1 mM EDTA; and 5% BSA). The DNA-protein complex was separated from the free probe in 4% native polyacrylamide gel and then dried and visualized by PhosphoImage analysis (Molecular Dynamics, Amersham). The specificity of NF-κB binding to DNA was confirmed by incubation with a double-stranded mutated oligonucleotide (Santa Cruz Biotechnology). In supershift assays, nuclear extracts were incubated with 0.5 µg of antibody against NF-κB subunit p65, p50, c-Rel, RelB or p52 (Santa Cruz Biotechnology) for 1 h on ice before incubation with the probe.

### Luciferase reporter assay

To evaluate NF-κB-dependent transcriptional activity, A549 cells were co-transfected with 770 ng p6κB-LUC (kindly provided by Dr. Patrick Baeuerle, University of Munich, Germany) and 30 ng pRL-CMV (Promega) plasmids using Lipofectamine 2000 reagent (Invitrogen). Transfected cells were infected for 24 hours, washed with PBS and lysed to measure firefly and Renilla luciferases using the Dual Luciferase Reporter System (Promega) and a TD-20/20 Luminometer (Turner Designs).

### IL-8 ELISA

The concentration of IL-8 in supernatants from A549 and HMEC-1 cultures infected for 21 hours was evaluated by ELISA, according to manufacturer's instruction (R&D Systems). Since ExoU kills a high percentage of PA103-infected cells, the number of viable cells was determined 1 hour post-infection by trypan blue exclusion assay and the results were reported as pg of IL-8 per 10^5^ cells.

### ChIP assay

ChIP assays were performed in A549 cells infected for 14 hours using the SimpleChIP Enzimatic Chromatin IP kit (Cell Signaling), according to manufacturer's instructions. Briefly, protein–DNA complexes were cross-linked with formaldehyde, cells were lysed and digested chromatins were obtained after Micrococcal Nuclease treatment and sonication. An aliquot of the total input was stored and 20 µg DNA of each cross-linked chromatin preparation were immunoprecipitated with 10 µg of anti-p65 antibody (Santa Cruz Biotechnology). A pool of all cross-linked chromatin preparations was immunoprecipitated with rabbit IgG isotype control to ensure specificity. After washing, chromatin-DNA cross-linking of immunoprecipitated and total input samples was reversed and DNA was purified. DNA samples were then amplified by semi-quantitative PCR and Real Time qPCR using the following primers against the NF-κB site in IL-8 promoter: 5′- ACT CAG GTT TGC CCT GAG GGG A -3′ (sense) and 5′- TGC CTT ATG GAG TGC TCC GGT G (antisense). The semi-quantitative PCR conditions were denaturation at 94°C for 2 min and 36 cycles of denaturation at 94°C for 45 sec, annealing at 55°C for 45 sec, and extension at 72°C for 30 sec. An additional extension step of 5 min at 72°C was carried out after the last cycle. PCR products were subjected to electrophoresis in a 2% agarose gel in TBE buffer and analyzed densitometrically.

### Mice infection

Female Swiss mice (8–12 weeks old) anesthetized with a mixture of ketamine (65 mg/kg) and xylazine (13 mg/kg) were infected intratracheally with 10^4^ colony-forming units of PA103 or PA103Δ*exoU* in 50 µL of lipopolysaccharide-free saline. As control, mice were instilled with saline only. To assess the role of NF-κB in ExoU-mediated proinflammatory response, mice were intraperitoneally inoculated with the NF-κB inhibitor Bay 11-7082 at 20 mg/kg, or DMSO (vehicle), 1 hour before bacterial infection. At 24 h post-infection, animals were euthanized by intraperitoneal injection of sodium pentobarbital and their lungs were flushed with 1 mL of PBS. All animal experiments were approved by the Animal Ethics Research Committee of the State University of Rio de Janeiro (protocol # CEUA/022/2011) and performed in accordance with the guidelines of this Committee.

### BALF analysis

Recovered BALF were assessed for KC concentration, using ELISA (R&D Systems). The concentration of total leukocytes was determined in a Neubauer chamber by trypan blue exclusion assay, whereas the number of neutrophils was determined by Diff-Quick staining method (Dade Diagnostic) followed by analysis of 100 cells in a light microscopy.

### Statistical analysis

Statistical significance was accepted at the P<0.05 level by one-way ANOVA for multiple group analysis with a Bonferroni adjustment.
